# Pioneering Community-Based Healthcare in Rural India: The Legacy of Dr. Rajnikant Arole and Dr. Mabelle Arole

**DOI:** 10.7759/cureus.67319

**Published:** 2024-08-20

**Authors:** Umesh Kawalkar, Amar Mankar, Manoj S Patil, Anshu Singh

**Affiliations:** 1 Community Medicine, Government Medical College (GMC) Akola, Akola, IND; 2 Community Medicine, Datta Meghe Institute of Higher Education and Research, Wardha, IND; 3 Research and Development, Jawaharlal Nehru Medical College, Datta Meghe Institute of Higher Education and Research, Wardha, IND

**Keywords:** community participation, public health and social work, historical vignette, village health workers, padma bhushan, rural health services

## Abstract

The landscape of healthcare in rural India has long been characterized by challenges that include accessibility, affordability, and cultural acceptance. Among these difficulties, the story of Dr. Rajnikant Arole and Dr. Mabelle Arole stands out as a beacon of hope and innovation. Their work in the rural areas of Maharashtra not only transformed the health outcomes of the local population but also set a precedent for community-based healthcare initiatives worldwide.

## Introduction and background

Early lives and inspirations

**Figure 1:**
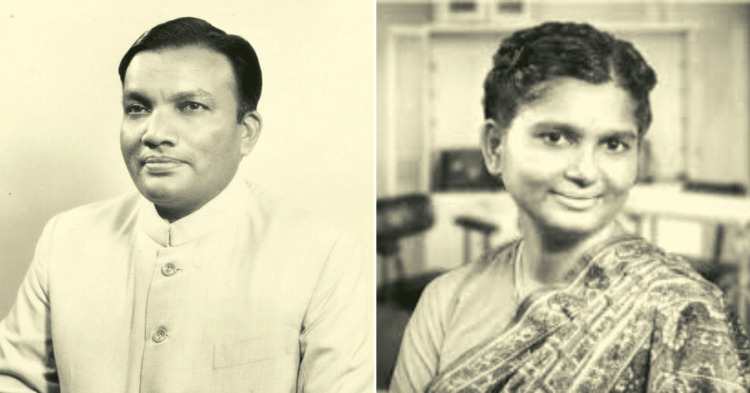
Dr. Rajnikant Arole (1934-2011) and Dr. Mabelle Arole (1935-1999). Image courtesy: Wikimedia Commons/Ramon Magsaysay Award Foundation.

Dr. Rajnikant Arole and Dr. Mabelle Arole (Figure 1), a team of husband and wife, were both driven by a deep-rooted commitment to social justice and public health. Dr. Rajnikant Arole was born into a family that valued education and service and pursued his medical degree with an ambition to address the medical inequalities in rural healthcare in India. Dr. Mabelle Arole, with her background in nursing and public health, shared a similar vision. Their meeting and subsequent partnership were marked by a mutual resolve to make a tangible difference in the lives of the underprivileged [1].

Genesis of the Jamkhed project

In the early 1970s, the Arole's chose Jamkhed, a drought-prone and socio-economically disadvantaged area in Maharashtra, as the site for their groundbreaking work. This decision was rooted in their belief that true healthcare extends beyond mere medical intervention; it encompasses the social, economic, and environmental determinants of health. The Comprehensive Rural Health Project (CRHP) they founded in 1970 aimed to empower the community by addressing these determinants through a holistic approach. Their vision was not just to treat diseases but to foster an environment where health could thrive through community engagement and education [2].

## Review

Innovative approaches and strategies

The Arole's approach was revolutionary for its time. They adopted a model that highlighted community participation, health education, and empowering women. Central to their strategy was the involvement of local communities in the planning and implementation of health initiatives. This participatory approach ensured that the interventions were culturally relevant and sustainable. By educating the community about basic health practices and preventive measures, the Arole's aimed to reduce the incidence of common illnesses. Health education sessions covered a range of topics, from sanitation and nutrition to maternal and child health. Recognizing the pivotal role women play in family health, the Arole's focused on training female health workers known as "Village Health Workers" (VHWs). These women, drawn from the community, received training in basic healthcare delivery, thus creating a sustainable network of local health providers. The CRHP model integrated preventive, promotive, and curative healthcare services. This comprehensive approach ensured that the community's healthcare needs were addressed at multiple levels, from primary care to more complex medical interventions [3,4]. Thus Arole's created a model that not only addressed immediate health concerns but also promoted long-term, sustainable development. The Arole's emphasized the importance of addressing the root causes of health issues, which often lay in poverty and lack of development. Their initiatives included income-generating activities, agricultural development, and vocational training to improve the overall quality of life in the community [5].

Achievements and impact

The impact of the CRHP was profound and multifaceted. Over the decades, the Arole's initiatives led to significant improvements in health outcomes. The reduction in infant and maternal mortality was particularly notable. Through targeted maternal and child health programs, the CRHP achieved dramatic reductions in infant and maternal mortality rates. The emphasis on antenatal care, safe childbirth practices, and neonatal care played a crucial role in this success [6]. The community-based approach helped in the early detection and treatment of infectious diseases such as tuberculosis and leprosy. The use of local health workers facilitated better compliance with treatment regimens and improved disease management. Nutritional education and the promotion of sanitary practices significantly reduced the incidence of malnutrition and waterborne diseases. Initiatives such as kitchen gardens and improved water sources contributed to these improvements. The empowerment of women through the VHW program had a ripple effect on the community. These women not only provided healthcare but also became a medium for education, sanitation, and women's rights, thereby fostering social change [7]. Despite their successes, the Arole's faced numerous challenges. The deeply entrenched socio-cultural norms, initial resistance from the community, and the sheer scale of rural healthcare needs were formidable obstacles. However, their resilience, adaptability, and unwavering commitment enabled them to overcome these challenges. They continuously refined their strategies based on feedback and evolving community needs, demonstrating the importance of flexibility in community health interventions [6].

Recognition and awards

Dr. Rajnikant Arole and Dr. Mabelle Arole's groundbreaking work did not go unnoticed. Over the years, they received numerous accolades that highlighted their contributions to healthcare and social development. Among the most prestigious awards was the Ramon Magsaysay Award in 1979, often considered the Nobel Prize of Asia, the award recognizing their remarkable contributions to community-based healthcare and their innovative approaches that empowered rural populations. They received the Padma Bhushan in 1990, one of India’s highest civilian awards, honouring their dedicated service and significant impact on rural health. In 2005, they received the Mother Teresa Award celebrating their humanitarian efforts and their enduring commitment to improving health outcomes for the poorest and most marginalized communities [1].

Legacy and continued relevance

The legacy of Dr. Rajnikant Arole and Dr. Mabelle Arole extends beyond Jamkhed. Their model has been studied and replicated in various parts of the world, influencing global health policies and practices. The principles of community participation, education, and empowerment remain central to many contemporary health initiatives. Their work also highlights the critical role of primary healthcare in achieving universal health coverage. By demonstrating that healthcare can be effectively delivered in resource-limited settings through community involvement and local capacity building, the Arole's set a blueprint for health equity. The Jamkhed project, rooted in the belief that health is a human right, has shown that even the most marginalized communities can achieve significant health improvements when they are empowered to take charge of their own health. This approach not only addresses immediate health needs but also builds the capacity of communities to sustain health improvements over the long term [6,7].

Key components of the Jamkhed model

The Jamkhed model is built on several key components that together create a comprehensive and sustainable approach to rural health care. Firstly, the model emphasizes the importance of primary health care, which includes basic curative services, preventive measures, and health education. This approach ensures that the most common health issues can be addressed at the community level, reducing the need for more costly and less accessible secondary and tertiary care. Secondly, the model focuses on training and empowering local health workers. The VHWs are central to this strategy. These women, selected by their communities, receive extensive training in basic health care, nutrition, sanitation, and maternal and child health. They serve as the first point of contact for health issues, providing education, treatment, and referral services. Their deep understanding of local culture and customs allows them to effectively communicate health messages and gain the trust of their communities. Thirdly, the model integrates health care with other aspects of development, recognizing that health cannot be improved in isolation. This includes initiatives in areas such as water and sanitation, agriculture, and income generation. By addressing the social determinants of health, the Jamkhed model creates an environment that supports overall well-being [2,4].

Educational and research contributions

Beyond their direct impact on healthcare delivery, the Arole's have also made significant contributions to health education and research. The CRHP has become a center for learning and innovation, attracting students, researchers, and health professionals from around the world. Through its training programs, workshops, and conferences, the CRHP disseminates the knowledge and experience gained from the Jamkhed project, fostering a new generation of health leaders committed to community-based care. The research conducted at CRHP has provided valuable insights into the effectiveness of community health interventions. Studies have documented the positive outcomes of the Jamkhed model, including reductions in infant and maternal mortality, improved management of chronic diseases, and increased community resilience. These findings have been published in academic journals and presented at international conferences, contributing to the evidence base for community health programs [4,5].

## Conclusions

The pioneering work of Dr. Rajnikant Arole and Dr. Mabelle Arole in Jamkhed stands as a powerful example of how community-based healthcare can transform lives and uplift entire communities. Their holistic approach, which integrated health care with broader social and economic development, addressed the root causes of health disparities and created sustainable improvements in health outcomes. The Jamkhed model, built on principles of community participation, empowerment, and comprehensive care, has had a lasting impact on global health practices. It continues to serve as a blueprint for achieving health equity and demonstrates the critical importance of addressing the social determinants of health. The legacy of the Arole's is a testament to the profound difference that dedicated individuals can make in the world, and their work remains a source of inspiration for future generations committed to health and social justice.
